# Psychiatric Burden in Chronic Sinonasal Diseases: A Single-Center Cross-Sectional Observational Study

**DOI:** 10.7759/cureus.57471

**Published:** 2024-04-02

**Authors:** Francesco Giombi, Luca Canali, Jessica Zuppardo, Gian Marco Pace, Francesca Pirola, Fabio Ferreli, Giuseppe Mercante, Giuseppe Spriano, Michele Cerasuolo, Luca Malvezzi

**Affiliations:** 1 Department of Biomedical Sciences, Humanitas University, Milan, ITA; 2 Otorhinolaryngology Unit, Humanitas Research Hospital, Milan, ITA; 3 Otorhinolaryngology Head and Neck Surgery Unit, Casa di Cura Humanitas San Pio X, Milan, ITA

**Keywords:** anxiety, depression, psychological impact, quality of life, septum deviation, chronic rhinosinusitis

## Abstract

Background and objectives: Chronic rhinosinusitis (CRS) and nasal septum deviation (SD) are two widely diffused clinical conditions in otorhinolaryngology clinical practice. Albeit nasal symptoms are the most commonly referred by patients affected by both conditions, recent evidence has explored the impairment of nasal function beyond its local implication. Indeed, the prevalence of psychiatric disorders, specifically anxiety and depression, was found higher in patients suffering from SD or CRS than in the general population. The aim of this study was to evaluate the psychiatric burden of these conditions in terms of anxiety and depression and to assess its relationship with clinical phenotype and age.

Methods: Monocentric cross-sectional observational study. Consecutive patients affected by CRS with or without nasal polyps or by SD were considered eligible. At referral, each patient underwent nasal endoscopy for clinical diagnosis and had to fill in the Hospital Anxiety and Depression Scale (HADS), the Sinonasal Outcome Test-22 (SNOT-22), and the Visual Analogue Scale (VAS) for global nasal symptoms. The population was grouped according to disease and age.

Results: One hundred fifty patients were enrolled. We observed a statistically significant difference in mean HADS score between patients affected by CRS with nasal polyps and those suffering from CRS without nasal polyps or SD both in the overall population and by age groups. Nevertheless, there was no significant difference in the HADS score between younger patients affected by CRS and SD. The mean HADS score was significantly higher in younger patients affected by SD compared to older. Furthermore, we observed an inverse correlation between age and HADS score in each disease group, statistically significant for SD. On the contrary, in the overall population, HADS score and patient-related outcomes (PROs) were directly correlated.

Conclusions: In the era of personalized medicine, our work remarks on the critical impact of anxiety and depression on the quality of life (QoL) of patients affected by sinonasal conditions. According to our results, age affects patient-reported outcomes (PROs) and should, therefore, be enhanced in the therapeutic decision process.

## Introduction

Chronic rhinosinusitis (CRS) and nasal septum deviation (SD) are two of the most common clinical conditions encountered in ENT practice. It is estimated that 14% to 16% of the adult population suffers from CRS, whether in its variants with nasal polyposis (CRSwNP) or without (CRSsNP), respectively [1-3]. In addition, according to the literature, up to 80% of the population has an SD even though up to 30% of patients endoscopically diagnosed with SD may not complain of nasal obstruction [4,5]. Both conditions carry a considerable impact on quality of life (QoL) and mental status, as shown by the fact that the prevalence of depression in CRS patients is estimated to be between 20% and 25% [6]. Similarly, in a recent meta-analysis, the prevalence of anxiety and depression disorders was found significantly higher in patients with SD compared to healthy controls [7]. Furthermore, recent studies have investigated the impairment of nasal function beyond its local implications, demonstrating its role in the development of systemic conditions such as obstructive sleep apnea syndrome (OSAS) and hypertension [8,9].

Although many studies have investigated the link between the severity of clinical-radiological profile and psychiatric comorbidities in patients with chronic sinonasal diseases, it is still a matter of debate whether these factors may be related. In a previous analysis, authors found no correlation between nasal symptoms and sinus opacification, regardless of possible psychiatric-associated conditions [10]. Conversely, Kara et al. observed a significant correlation between patient-reported rhinology outcomes (e.g., Sinonasal Outcome Test-22 (SNOT-22)) and anxiety and depression [11]. Likewise, even within the context of nasal septal deviation, a clear relationship between objective clinical assessment and subjective psychometric evaluation remains elusive. In this regard, a recent analysis showed that, between several different patient-reported outcomes (PROs), only a few were significantly associated with post-operative QoL (e.g., nasal obstruction symptom severity, sleep quality, and stress levels) [12].

Over the years, different tools have been developed to measure the impact of disease on patients’ QoL and/or mental well-being [13]. One of them, the Hospital Anxiety and Depression Scale (HADS), was originally designed by Zigmond and Snaith to capture anxiety and/or depression in non-psychiatric settings [14]. This rapid self-reported questionnaire has been translated into many languages and to date it is also validated to assess the psychological status of patients suffering from CRS [15].

The aim of the present study was to measure anxiety and depression in patients affected by chronic sinonasal diseases, to assess possible differences based on the specific clinical phenotypes, and to evaluate the correlation between HADS score and age in each clinical condition.

## Materials and methods

This study is a single-center cross-sectional observational study. All adult patients who were subsequently referred to the Otolaryngology Clinic at IRCCS Humanitas Research Hospital (Milan, Italy) between March 2021 and December 2021 for SD, CRSsNP, or CRSwNP were enrolled.

The study was conducted in accordance with the ethical standards of the Declaration of Helsinki and its later amendments, and it was approved by the ethical committee of our institute (IRCCS-ICH-IEC/3114). All included patients gave their informed consent.

The diagnosis of SD or CRS was done at the patients’ referral, with the endoscopic examination routinely performed in the outpatient clinic and CT scan findings when necessary, according to the guidelines [16]. As recently stated in the last European Position Paper on Rhinosinusitis and Nasal Polyps (EPOS 2020), CRS was diagnosed in case of long-lasting (>12 weeks) inflammation of the nasal cavity and paranasal sinuses, characterized by symptoms of nasal blockage/congestion or nasal discharge, possibly associated to facial pain/pressure and a dysregulated sense of smell [16]. Data about patients’ history were collected from medical records. According to standard clinical practice in our academic department and to ensure a personalized approach, patients referring to sinonasal symptoms were evaluated by different specialists (allergologist, pneumologist, and rhinologist) using a multidisciplinary approach. Patients who were under allergen-specific immunotherapy were not considered eligible, since it may impact nasal symptoms, thus providing selection bias [17]. Also, any patient who revealed cognitive impairment (age- or disease-related, or under medications for psychiatric conditions) at the Quick Mild Cognitive Impairment (QMCI) test-I was excluded [18]. Atopy was defined as sensitization to at least one inhalant allergen. First-line treatment was composed of topical medication, namely daily rinses with saline solution multiple times a day, as well as steroid nebulization twice a day (budesonide: 0.5 mg/mL).

Patients were sub-grouped according to disease (e.g., SD, CRSsNP, and CRSwNP) and age (median). During the examination, each patient was asked to complete the Italian version of the HADS questionnaire, the SNOT-22, and Visual Analogue Scales (VAS) for sinonasal symptoms, in order to assess PROs [14,19-21]. The global SNOT-22 score gives an indication of the impact of the symptoms on patients’ QoL [20]. Likewise, global VAS is meant to address the question “How bad is my nasal condition overall?” [21].

Results were anonymously collected and archived in a customized Excel® (Microsoft Corp, Seattle, Washington, USA) spreadsheet. All the statistical analyses were performed using SPSS Statistics for Macintosh, Version 28.0 (IBM Corp., Armonk, NY). Ordinal variables were expressed by numbers and percentages, whereas continuous parametrical data were presented by mean and range. Normal distribution was ascertained through the Shapiro-Wilk test. Significant differences were determined with a standard 0.05 alpha level (p-value), which was adjusted using Bonferroni correction in case of multiple comparisons. Differences between continuous parametrical variables were calculated with student t-tests or through one-way analysis of variance (ANOVA) in cases when more than two groups were compared. Pearson coefficient (p) was used to estimate correlation. A p-value <0.05 was considered necessary for statistical significance.

## Results

Overall, 150 patients referred to the otorhinolaryngology department in Humanitas, Rozzano (Milan) between January 2020 and December 2022 were enrolled in the study. There were 79 (52.67%) males and 71 (47.33%) females. Thirty-eight (25.33%) patients were affected by SD, 49 (32.67%) by CRSsNP, and 63 (42.0%) by CRSwNP. Ninety-eight (65.33%) patients were atopic (e.g., sensitized to at least one inhalant allergen) and 45 (30.0%) previously received a diagnosis of asthma. The mean age was 46.16±13.18 years (median: 46; range: 18-77). Sociodemographic characteristics are resumed in Table 1. Results were normally distributed according to the Shapiro-Wilk test (p-value >0.05).

**Table 1:** Sociodemographic characteristics of the included patients sd, standard deviation; CRSwNP, chronic rhinosinusitis with nasal polyps; CRSsNP: chronic rhinosinusitis without nasal polyps; SD: septum deviation

Gender	Females (%)	71 (43.33)
	Males (%)	79 (52.67)
Age	Mean±sd	46.16±13.18
Body mass index	Kg/m^2^	27.12
Smoking	Yes (%)	35 (23.33)
	No (%)	115 (76.67)
Sinonasal condition	CRSwNP (%)	63 (42.0)
	CRSsNP (%)	49 (32.67)
	SD (%)	38 (25.33)
Comorbidities	Asthma (%)	45 (30.0)
	Allergy (%)	98 (63.33)

Overall, the mean HADS score was 10.96±7.92 (median: 10; range: 0-42); HADS score by disease: SD=6.36±5.16; CRS=11.21±7.39 (CRSsNP=7.74±5.06, CRSwNP=13.81±7.82). HADS scores and PROs in the overall population and by age subgroups are displayed in Table 2.

**Table 2:** Mean HADS score, anxiety- and depression-related items, and PROs in the overall population and by age subgroups sd, standard deviation; CRS, chronic rhinosinusitis; CRSwNP, chronic rhinosinusitis with nasal polyps; CRSsNP, chronic rhinosinusitis without nasal polyps; SD, septum deviation; HADS, Hospital Anxiety and Depression Scale; SNOT, Sinonasal Outcome Test; VAS, Visual Analogue Scale; PROs, patient-reported outcomes

			HADS±sd (range 0-42)	HADS-anxiety±sd (range 0-21)	HADS-depression±sd (range 0-21)	SNOT-22±sd (range 0-110)	VAS-global±sd (range 0-10)
Overall	CRS							11.21±7.39	6.85±4.13	4.36±3.82	26.32±10.32	6.17±1.46
		CRSsNP	7.74±5.06	5.29±3.58	2.44±1.97	19.57±7.40	5.64±1.58
		CRSwNP	13.81±7.82	8.01±4.14	5.79±4.24	31.35±9.31	6.54±1.24
	SD		6.36±5.16	4.38±3.64	1.97±2.02	7.53±3.62	4.69±1.80
>46 years	CRS		11.84±7.79	7.00±4.27	4.84±4.05	28.42±8.92	6.32±1.51
		CRSsNP	7.68±4.23	5.42±3.02	2.26±1.85	22.11±7.09	5.95±1.90
		CRSwNP	13.82±8.34	7.75±4.60	6.08±4.23	31.42±8.14	6.50±1.28
	SD		3.82±3.37	3.00±2.75	0.81±1.32	8.27±3.84	5.09±1.70
<46 years	CRS		10.73±6.99	6.85±4.02	3.88±3.59	23.42±11.03	5.94±1.35
		CRSsNP	7.88±5.71	5.29±4.02	2.59±2.11	17.59±7.21	5.44±1.34
		CRSwNP	14.38±6.88	8.85±3.05	5.52±4.40	30.90±10.66	6.57±1.12
	SD		7.75±5.32	5.21±3.79	2.54±2.10	7.29±3.59	4.58±1.86

In >46-year-old population, depression-related scores were significantly higher than in <46 year old (difference of the means: 0.85, p-value=0.036), whereas no significance was observed for anxiety-related items between age subgroups (Table 3).

**Table 3:** Difference of anxiety and depression-related items by age subgroups HADS, Hospital Anxiety and Depression Scale

HADS items	Age (years)	n	Mean	Difference (CI 95%)	p-value
Overall	<46	76	9.50±6.59	1.09 (-3.45-1.27)	0.360
	>46	74	10.59±7.83		
Anxiety	<46	76	6.13±4.00	0.24 (-1.60-1.12)	0.920
	>46	74	6.37±4.31		
Depression	<46	76	3.37±3.16	0.85 (-2.02-0.33)	0.036
	>46	74	4.21±4.02		

Overall, we observed a statistically significant difference in mean HADS score between patients affected by CRSwNP and those who suffered from CRSsNP or SD (6.06, p<0.001; 7.45, p<0.001, respectively), although there was no significance comparing CRSsNP and SD (1.38, p=0.856; Table 4). Regarding PROs, we also observed significant results (p-value <0.05) by comparing the average SNOT-22 scores in each clinical condition and by age subgroups (Table 4). Correlation analysis between the HADS score and PROs resulted is statistically significant (Pearson coefficient: HADS-SNOT-22=0.386, p-value <0.001; HADS - global-VAS=0.285, p-value <0.001). Analysis of variance showed a significantly different distribution of HADS, SNOT-22, and global-VAS scores between aggregate disease subgroups, in both the overall population and age-pooled samples (p-value <0.001). In age-grouped analysis, the difference in mean HADS score by disease was still significant between CRSwNP and both CRSsNP and SD, although quantitatively lower in <46-year-old population compared to >46 year old (CRSwNP vs SD in >46 year old: 10.01, in <46 year old: 6.63; Table 4). Furthermore, in <46-year-old group, the difference in mean HADS score was not significant between patients affected by CRSsNP and SD (0.14, p=0.942); conversely, in >46-year-old patients affected by CRSsNP, mean HADS score observed had a statistically significant difference compared to those suffering from SD (3.86, p=0.024, Table 4).

**Table 4:** Difference of mean scores in overall population and by age subgroups CRS, chronic rhinosinusitis; CRSwNP, chronic rhinosinusitis with nasal polyps; CRSwNP, chronic rhinosinusitis without nasal polyps; SD, septal deviation; HADS, Hospital Anxiety and Depression Scale; SNOT, Sinonasal Outcome Test; VAS, Visual Analogue Scale

	Disease		HADS (CI 95%)	p-value	SNOT-22 (CI 95%)	p-value	Global-VAS	p-value
Overall									CRS	vs SD	4.86 (2.23-7.48)	<0.001	18.79 (15.31-22.26)	<0.001	1.48 (0.88-2.07)	<0.001
	in CRSwNP	vs SD	7.45 (4.55-10.34)	<0.001	23.82 (20.61-27.04)	<0.001	1.87 (1.20-2.56)	<0.001
		vs CRSsNP	6.06 (3.47-8.65)	<0.001	11.77 (8.51-15.04)	<0.001	0.93 (0.40-1.47)	0.004
	in CRSsNP	vs SD	1.38 (-3.63-0.86)	0.856	12.04 (9.37-14.72)	0.002	0.94 (0.20-1.68)	0.026
>46 years	CRS	vs SD	8.02 (3.23-12.82)	0.001	20.15 (14.66-20.15)	<0.001	1.23 (0.22-2.24)	0.023
	In CRSwNP	vs SD	10.01 (4.81-15.20)	<0.001	23.15 (18.03-28.26)	<0.001	1.41 (0.47-2.35)	0.004
		vs CRSsNP	6.14 (2.06-10.21)	0.012	9.32 (4.95-13.68)	<0.001	0.55 (-0.29-1.39)	0.193
	In CRSsNP	vs SD	3.86 (0.80-6.92)	0.024	13.83 (9.07-18.59)	0.004	0.86 (-0.56-2.28)	0.227
<46 years	CRS	vs SD	2.98 (-6.23-0.27)	0.091	16.13 (11.50-20.75)	<0.001	1.35 (0.59-2.12)	0.002
	In CRSwNP	vs SD	6.63 (2.93-10.33)	0.004	23.61 (18.95-28.27)	<0.001	1.99 (1.05-2.93)	<0.001
		vs CRSsNP	6.49 (2.83-10.15)	0.002	13.31 (8.11-18.51)	<0.001	1.13 (0.39-1.86)	0.003
	In CRSsNP	vs SD	0.14 (-3.28-3.00)	0.942	10.30 (7.03-13.57)	<0.001	0.86 (-0.04-1.77)	0.143

This evidence reflects the significant differences in mean HADS score between <46-year-old and >46-year-old populations affected by SD (difference of the mean: 3.66, p-value=0.045), whereas no significance was observed between the other variables (Table 5).

**Table 5:** Difference of mean scores between age subgroups CRS, chronic rhinosinusitis; CRSwNP, chronic rhinosinusitis with nasal polyps; CRSwNP, chronic rhinosinusitis without nasal polyps; SD, septal deviation; HADS, Hospital Anxiety and Depression Scale; SNOT, Sinonasal Outcome Test; VAS, Visual Analogue Scale

		Difference mean scores (p-value)		
		>46 years		
<46 years		HADS (range 0-42)	SNOT-22 (range 0-110)	VAS-global (range 0-0)
	CRSwNP	-0.04 (0.984)	-0.21 (0.933)	0.20 (0.543)
	CRSsNP	0.10 (0.947)	-4.25 (0.052)	- 0.52 (0.274)
	SD	3.66 (0.045)	-1.07 (0.441)	- 0.57 (0.389)

Finally, Pearson coefficient calculation showed an inverse correlation between age and HADS score in each disease group, higher and statistically significant for SD and CRSwNP (CRSsNP=-0.27, p=0.057; CRSwNP=-0.29, p=0.024; SD=-0.32, p=0.045; Table 6).

**Table 6:** Correlation between age and PROs. Results are reported in terms of the Pearson coefficient CRS, chronic rhinosinusitis; CRSwNP, chronic rhinosinusitis with nasal polyps; CRSwNP, chronic rhinosinusitis without nasal polyps; SD, septal deviation; HADS, Hospital Anxiety and Depression Scale; SNOT, Sinonasal Outcome Test; VAS, Visual Analogue Scale

		HADS	p-value	SNOT-22	p-value	VAS-global	p-value
Overall		-0.04	0.329	0.18	0.213	0.21	0.129
CRS		-0.12	0.110	0.22	0.093	0.18	0.111
	CRSsNP	-0.27	0.057	0.18	0.147	0.12	0.195
	CRSwNP	-0.29	0.024	0.24	0.098	0.21	0.093
SD		-0.32	0.045	0.15	0.174	0.13	0.183

## Discussion

Functional nasal diseases, such as SD and CRS with or without nasal polyps, are chronic conditions and, as such, need to be addressed not only from a merely clinical point of view but also from a psychological perspective. The prevalence of depression and anxiety in patients affected by CRS has been estimated to be between 25.2% and 28.9%, respectively, in a large meta-analysis covering 40,956 patients, highlighting the significance of this issue [22]. A recent nationwide South Korean study measured an overall incidence of depression 1.51-fold higher in the CRS group compared to controls [23]. Similarly, Choi HG et al. estimated a significantly increased risk of depression in patients affected by CRS rather than in healthy subjects (adjusted hazard ratio=1.41) [24]. Furthermore, Qiang M et al. found that depression and anxiety, as well as the co-morbidity of depression with anxiety, were more common in patients affected by nasal SD in comparison to controls (39.5% vs 22.8%, p=0.025; 38.2% vs 15.2%, p=0.001; and 27.6% vs 11.4%, p=0.011, respectively) [25].

Several studies have already explored the association between reported symptoms and psychometric questionnaires. Phillips KM et al. found a correlation between Nasal Obstruction Symptom Evaluation (NOSE) and a 2-item Patient Health Questionnaire (PHQ-2) in patients affected by CRS [26]. Similarly, Tomoum M et al. assessed the interrelation between the Rhinosinusitis Disability Index (RSDI), which analyses the disease-related impact on QoL, and depression/anxiety score as determined by HADS [15]. Moreover, the SNOT-22, which is validated for both CRS and SD patients, has been also found to strictly correlate with HADS, in both total and psychiatric domain-specific scores [27,28].

In this context, our study is consistent with previously reported data. The overall average HADS score in our series was higher than those previously reported in the general population, confirming the severe burden of depression and anxiety on chronic sinonasal conditions [29,30]. In patients who were diagnosed with CRSwNP, we observed a higher HADS score than both CRSsNP and SD, regardless of age subgroups (Table 4). This difference reflects what has already been published in the literature, being consistent with recent research by Vogt et al., who reported a slightly higher prevalence of anxiety (53.06% vs 45.66%) and depression (40.82% vs 36.95%) stratifying CRS patients by the presence of nasal polyps [31]. Notably, this is the first reported series comparing HADS scores within CRSw/sNP and SD groups. Overall, those who suffered from CRS showed a higher HADS score than patients with SD (Table 4). It is likely that, in the presence of a complex clinical picture, consisting of symptoms overwhelming the mere nasal obstruction such as in CRS, patients may experience a more severe impairment of psychiatric domains such as anxiety and depression. As expected, we also observed a positive correlation between HADS score and PROs (p<0.001) as well as significant differences in PROs between each clinical condition (p<0.05; Table 4). These results confirm that the endoscopic and symptomatological differences may further turn into divergences from a psychiatric point of view, in terms of depression and anxiety. According to our evidence, we want to stress the need for a change from a “disease” to a “patient-centered” medicine, in order to improve the whole healthcare process from a holistic perspective [32].

Furthermore, we particularly focused on the relationship between functional nasal conditions and age. As already proposed in published literature, our population was divided into numerically equal groups based on median age, and outcomes in different age subgroups were directly compared [33]. Depression-related items were significantly higher in older (e.g., >46 years old) patients, whereas no difference was observed for anxiety-related items (Table 3). This could be explained by the different age of onset of these two conditions. De la Torre et al. reported the age patterns of depression in a large sample of patients (n=17152), observing a significantly higher prevalence of depressive symptoms in those aged 45 to 59 years old compared with those aged 16 to 29 [34]. Conversely, as concerns anxiety-spectrum disorders, a recent meta-analysis found a mean age of onset of 21.3 years [35].

As opposed to the overall population, post-hoc analysis in age-pooled samples showed no significant difference in HADS score between CRS and SD in younger patients (e.g., <46 year old; Table 4). Moreover, among all variables, the difference by age subgroups was statistically significant only for SD (3.66, p=0.047), whereas no significance was observed for CRSs/wNP as well as for the other PROs (Table 5). Finally, we observed a statistically significant inverse correlation between age and HADS score in patients diagnosed with SD (p=-0.32, p=0.045) and CRSwNP (p=-0.29, p=0.024; Table 6). This lets us speculate that functional nasal disorders, despite having a global symptomatologic burden that progresses with age, present a significant impact in the psychiatric sphere of younger patients. In the literature, no association between age and depression or anxiety has been previously found in patients affected by chronic sinonasal conditions [5,36,37]. According to our results, the HADS score is inversely related to age, and this correlation was higher for generally less symptomatic conditions such as SD rather than CRSs/wNP.

This study is subject to several limitations. First, its cross-sectional observational design is susceptible to various potential biases. The analyzed population may not represent the general population, and confounding factors may not have been fully considered, thus limiting the widespread applicability of our results. Additionally, since the study analyzes anxiety and depression burden at a single specific time point (before surgery), further analysis of changes over time, particularly after surgery, was not possible. Prospective studies are warranted to better understand the impact of surgery on these parameters. Concerning PROs, in our analysis we only included global scores without considering the significance of each single SNOT-22- or VAS-related item. Herein, since the reliability of PROs has already been demonstrated in the literature, we primarily aimed to focus the attention on anxiety- and depression-related outcomes. Furthermore, our investigation was restricted to two specific clinical conditions, and we only examined depression and anxiety using the HADS score. Several other factors, including sociodemographic characteristics, treatment modalities, and disease-specific issues, were not explored, thereby limiting the comprehensiveness of our psychiatric assessment. These limitations were imposed by intrinsic feasibility constraints (e.g., the limited amount of Italian-validated psychometric scores) and a desire to maintain methodological consistency throughout the study. However, future multicentric prospective studies should aim to broaden the assessment of psychiatric burdens across the diverse spectrum of sinonasal diseases.

Within the context of the emerging era of personalized medicine, our results, considering the aforementioned limitations, could represent another piece of the puzzle in future developments toward the accurate stratification of patients suffering from sinonasal diseases. This extends beyond the sole phenotypic and endotypic definitions of disease, incorporating potential implications of the psychological sphere of patients, particularly focusing on anxiety and depression.

## Conclusions

Our study analyzes the burden of the most widespread functional sinonasal diseases upon patients’ depressive and anxious state, by using the HADS questionnaire. As mostly expected, CRSwNP proved to be the most impactful condition; however, SD demonstrated the highest correlation with age. In particular, we observed an inverse correlation between the HADS score and the age of the cohort, suggesting that sinonasal functional pathology may have a greater impact on the younger population (<46 years).

In surgical medical contexts, the impact of anxiety- and depression-related factors on QoL is frequently underrated. Our article aims to emphasize the importance of addressing these factors to reach a more comprehensive management of patients with sinonasal disorders. This approach involves considering anxiety and depression alongside physical symptoms, promoting a multidisciplinary and targeted strategy to enhance outcomes for all patients.
